# Editorial: Advances in neuroimaging of epilepsy

**DOI:** 10.3389/fneur.2023.1142503

**Published:** 2023-02-07

**Authors:** Emanuele Bartolini, Lorenzo Caciagli, Sara Larivière, Karin Trimmel

**Affiliations:** ^1^IRCCS Stella Maris Foundation, Department of Developmental Neuroscience, Pisa, Italy; ^2^Tuscany PhD Programme in Neurosciences, Florence, Italy; ^3^Department of Bioengineering, University of Pennsylvania, Philadelphia, PA, United States; ^4^Center for Brain Circuit Therapeutics, Brigham and Women's Hospital and Harvard Medical School, Boston, MA, United States; ^5^Department of Neurology, Medical University of Vienna, Vienna, Austria

**Keywords:** seizures, brain morphometry, functional connectivity, quantitative imaging, MRI

Epilepsy is a multifaceted disorder that can have a variety of etiologic factors, including genetic and structural causes. Neuroimaging is an essential component of its diagnostic workup. Conventional MRI scans can reveal visible structural brain abnormalities that may be the cause of seizures in both common and rare forms of epilepsy. However, in some cases, even when the suspicion of an epileptogenic structural lesion is high, such as in patients being referred for epilepsy surgery, a conventional brain MRI may not reveal any abnormalities. Advanced neuroimaging techniques such as MRI fingerprinting, brain morphometry, functional MRI, and ultra-high field MRI can provide additional diagnostic information and can help identify subtle or cryptic lesions that may not be visible on a conventional brain MRI. This can aid in more accurate clinical assessments of individuals with epilepsy, or those with an unclear etiology. Advanced neuroimaging is a powerful tool to investigate neural networks underlying seizure generation, epilepsy-associated large-scale system reorganization, and substrates of cognitive comorbidities. Most advanced imaging techniques can be applied to study both groups and single subjects, providing working hypotheses that can subsequently be explored in larger studies. Comprehensive views can be further provided by multisite neuroimaging meta-analyses, that explore morphological and functional brain abnormalities shared by subjects with common epilepsies or specific syndromes.

The aim of this Research Topic is to give an in-depth overview of the latest advances in epilepsy research that have been made using diverse and innovative neuroimaging techniques. Our focus has been 2-fold: (i) Hypothesis testing and pursuit of mechanisms: how advanced neuroimaging can be employed to verify or explore the pathophysiological processes underlying common epilepsies and (ii) Clinical application: to define the role of advanced neuroimaging techniques in the diagnostic workup of both common and rare epilepsies.

With this purpose, we called for articles focusing on: identification of subtle morphological brain abnormalities *via* conventional neuroimaging approaches; investigation on the microscopic characteristics of brain lesions identified by conventional imaging; analysis of structural and functional brain abnormalities in specific syndromes or across the epilepsy spectrum; exploration of the functional networks associated with seizures/interictal spikes, epilepsy-related neural system reorganization, and cognitive comorbidities.

Upon our call for papers, five high-quality manuscripts have been submitted and underwent peer-review. Three papers met the standards we deemed adequate for this topic and we gathered two additional articles of particular value. Pizzanelli et al. proposed a functional connectivity study to investigate a group of adults with drug-sensitive temporal lobe epilepsy (i.e., “benign”) by means of 3 Tesla resting-state functional MRI. They showed that reorganization of mesiotemporal functional connectivity can be observed also in seizure-free patients and that lateralization-related differences are remarkable. He et al. analyzed hippocampal malrotation with qualitative and quantitative parameters in adults with focal cortical dysplasia, disclosing no differences between patients and healthy controls. Zhang et al. explored brain morphometry of young patients with juvenile myoclonic epilepsy, showing widespread microscopic structural abnormalities associated with cognitive performances and disease course. Machegger et al. showed that quantitative analysis of diffusion restriction and ADC decrease on MRI is applicable to differentiate acute ischemic stroke from status epilepticus with high sensitivity and specificity. Lastly, Fukuma et al. developed a novel asymmetry method to localize areas of hyperperfusion in poststroke epilepsy using a single postictal single-photon emission computed tomography (SPECT) study. While the gold standard for localizing epileptogenic seizures in epilepsy is subtraction imaging of ictal and interictal perfusion SPECT coregistered to MRI (SISCOM), in many clinical settings, ictal perfusion studies are difficult to obtain, and postictal studies are used instead. Comparing a standardized asymmetry analysis vs. postictal—interictal subtraction of perfusion SPECT images, Fukuma et al. demonstrated that a single scan postictal SPECT asymmetry analysis may offer diagnostic results in certain cases, at the cost of a sensitivity reduced by 20%, which may be still complemented by the subtraction method in unclear cases. In certain clinical settings, such a stepwise procedure could result in reducing the number of scans needed for seizure focus localization, limiting radiation exposure, and scanning costs.

The quality of these selected articles was remarkable and allowed us to provide an overview on different and up-to-date techniques in advanced neuroimaging in epilepsy. One of the most relevant themes of this collection is the breadth of imaging-driven information that can be applied to categorize individuals with epilepsy. The widespread use of non-invasive biomarkers that might assist in predicting seizure outcome and associated comorbidities and to tailor epilepsy surgery would in fact be of utmost importance. We are confident that our community will strongly pursue this quest to improve the life and care of people with epilepsy.

## Author contributions

All authors listed have made a substantial, direct, and intellectual contribution to the work and approved it for publication.

